# DEVELOPMENT OF THE SELF-EFFICACY IN SMARTPHONE USE SCALE FOR OLDER ADULTS

**DOI:** 10.1093/geroni/igae098.3546

**Published:** 2024-12-31

**Authors:** Yutian Cheng, Yasuyuki Gondo

**Affiliations:** Osaka University, Suita, Osaka, Japan; Osaka University, Suita, Osaka, Japan

## Abstract

Currently, a large proportion of older adults are still unable to use smartphone functions, except for calling or dialing. Considering the strategy to accelerate smartphone use, developing a tool to assess users’ confidence is important for future intervention studies. Thus, this study aimed to develop the scale for measuring the self-efficacy of smartphone use for older adults. We came up with 38 items first, derived former researches and some of developed own experience from smartphone lecture held for older adults. We conducted an internet survey for Japanese older adults aged 60 to 89, totally 400 participants (50% are female). Exploratory factor analysis and correlation analysis with general self-efficacy were conducted. By deleting those items which factor loading was too low or correlation with general analysis was not significant, the final questionnaire turned out to be 26 items. Further factor analysis revealed a three-factorial solution with good reliability (Cronbach alpha is 0.926). Three factors are named as Confidence (Cronbach alpha is 0.928), Problem Solving (Cronbach alpha is 0.904), and Realization of Benefit (Cronbach alpha is 0.855). Scores of self-efficacy of smartphone use showed significant positive correlations between general self-efficacy, which means high level of construct validity. With the development of this scale, it found out to be more than one dimension of self-efficacy in smartphone use, provided more precise assessment for further study. In further application research, we hope to develop a shorter version so it can reduce older adults’ burden.

